# Ghrelin, via corticotropin‐releasing factor receptors, reduces glucose uptake and increases lipid content in mouse myoblasts cells

**DOI:** 10.14814/phy2.14654

**Published:** 2021-01-19

**Authors:** Michal Elbaz, Eran Gershon

**Affiliations:** ^1^ Department of Ruminant Science Agricultural Research Organization Rishon LeZion Israel

**Keywords:** C2C12 cell, CRF receptor, des‐acyl ghrelin, glucose metabolism, lipid metabolism

## Abstract

Ghrelin and the corticotropin‐releasing factor (CRF) family are known regulators of cellular metabolism and energy balance. We previously demonstrated that myoblast glucose metabolism is regulated by ghrelin and that this effect is mediated by CRF receptor type 2 (CRF‐R2). Here we explored the effect of des‐acyl ghrelin, the major circulating isoform of ghrelin, on cellular metabolism in mouse myoblast C2C12 cells, and examined whether CRF family receptors mediate its metabolic effects in muscle cells. C2C12 cells were exposed to des‐acyl ghrelin with or without the CRF‐R1‐ and CRF‐R2‐specific antagonists antalarmin or antisauvagine‐30, respectively. Des‐acyl ghrelin reduced glucose uptake and expression of the glucose transporter GLUT4, but induced retinol‐binding protein 4 (RBP4) expression. Antalarmin and antisauvagine‐30 inhibited the induction of glucose uptake by des‐acyl ghrelin and its effect on GLUT4 and RBP4 expression. Moreover, treating C2C12 cells with des‐acyl ghrelin resulted in cAMP activation in response to the CRF‐R1‐specific ligand stressin, and the CRF‐R2‐specific ligand Ucn3. Furthermore, des‐acyl ghrelin reduced the expression of uncoupling proteins UCP2 and UCP3. Adding antalarmin or antisauvagine‐30 to the medium reversed this effect. Finally, des‐acyl ghrelin elevated lipid content and acetyl‐CoA carboxylase expression in C2C12 cells. Our results suggest that during food deprivation, des‐acyl ghrelin signals the muscle cells that glucose levels are low and that they should switch to fatty acids for their metabolic fuel.

## Graphical abstract

Des acyl‐ghrelin regulates glucose and lipids metabolism in C2C12 cells. Des acyl‐ghrelin elevates cAMP levels in response to stressin or UCN3 in C2C12 cells. CRF receptors type 1 and type 2 mediate des acyl‐ghrelin activity in C2C12 cells.

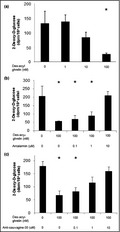

## 1 INTRODUCTION

The gut–brain peptide ghrelin has a known role in controlling growth hormone secretion as well as appetite (Kojima et al., 1999). Two isoforms of ghrelin are produced in the body: one is acylated on serine in position 3 with an O‐*n*‐octanoylatylgroup and is termed ghrelin, and the other is des‐acyl ghrelin, which lacks this O‐*n*‐octanoylatyl group (Hosoda et al., 2006). Both isoforms of ghrelin control many physiological processes, such as metabolic regulation in different cell types, including muscle cells (Barazzoni et al., 2005, 2007; Delhanty et al., 2010; Heijboer et al., 2006; Meier & Gressner, 2004; Vestergaard et al., 2011).

The effects of des‐acyl ghrelin on muscles have been widely studied. An in vitro study in C2C12 skeletal myoblasts showed that ghrelin, as well as des‐acyl ghrelin, stimulate differentiation, proliferation, and fusion into multinucleated myotubes through the activation of p38 (Filigheddu et al., 2007). Another study showed that des‐acyl ghrelin abolishes TNFα and interferon (INF)γ inhibition of protein synthesis in C2C12 cells (Sheriff et al., 2012). That study further demonstrated that des‐acyl ghrelin attenuates the atrophy signal upregulated by TNFα + IFNγ in C2C12 myotubes (Sheriff et al., 2012). It was further shown that in rats, des‐acyl ghrelin reduces mitochondrial reactive oxygen species (ROS) generation and inflammatory cytokine production while enhancing insulin‐stimulated glucose uptake (Gortan Cappellari et al., 2016). In myotubes, des‐acyl ghrelin lowered mitochondrial ROS production and enhanced insulin signaling (Gortan Cappellari et al., 2016). Both isoforms of ghrelin were further shown to increase fatty acid oxidation in rat soleus muscle in vitro (Cervone et al., 2020; Kraft et al., 2019). Des‐acyl ghrelin was also shown to conserve glucose uptake in isolated mature rat skeletal muscle exposed to fatty acids (Cervone et al., 2020).

In addition to ghrelin isoforms, the corticotropin‐releasing factor (CRF) family of peptides and receptors (Chen et al., 1993; Lovenberg et al., 1995; Perrin et al., 1995; Vale et al., 1981) has also been shown to control appetite and skeletal muscle energy balance. Levels of CRF receptor type 2 (CRF‐R2) are elevated by high‐fat feeding and chronic variable stress conditions in skeletal muscle (Kuperman et al., 2011). These conditions are also related to muscle insulin resistance (Corcoran et al., 2007; Hung & Ikizler, 2011; Kewalramani et al., 2010; Li et al., 2013; Martins et al., 2012; Mei et al., 2011). Another study demonstrated that in C2C12 myotubes, CRF‐R2 suppression of insulin‐induced glucose uptake is mediated by cAMP (Chao et al., 2015). Moreover, stimulation of CRF‐R2 by (its ligand?) Ucn2 in C2C12 myotubes attenuated phosphorylation of Akt and inhibited insulin‐induced glucose uptake (Chen et al., 2006). These results suggest that CRF‐R2 signaling is involved in reducing insulin action in muscles under stress or high‐fat diet conditions.

An association between the CRF family and des‐acyl ghrelin has been previously demonstrated. In rats, the effect of des‐acyl ghrelin on gastric motility was reversed by the local administration of a CRF‐R2 antagonist (Chen et al., 2005). Another paper supported these findings by showing that des‐acyl ghrelin inhibits antral motility, and that this effect may be mediated by CRF‐R2 in the brain (Fujimiya et al., 2012). A recent paper further supported these data by demonstrating that reduction in CRF‐R2 in the soleus muscle of rats fed a high‐fat diet for 6 weeks leads to a reduction in the palmitate oxidation induced by ghrelin stimulation (Cervone et al., 2020).

We previously found that in C2C12 cells exposed to ghrelin, the glucose transport protein GLUT4 is translocated to the cell membrane and expression of the adipokine retinol‐binding protein 4 (RBP4) is reduced, resulting in increased uptake of glucose by the cells. We further demonstrated that ghrelin upregulates only CRF‐R2 expression, whereas des‐acyl ghrelin induces the expression of both CRF‐R1 and CRF‐R2. In addition, we showed that the effects of ghrelin can be reversed by CRF‐R2 antagonist (Gershon & Vale, 2014). The expression of the key regulators of cell homeostasis—*UCP2* and *UCP3*—also increased upon exposure of the cells to ghrelin, and this effect could be blocked by the CRF‐R2 antagonist antisauvagine‐30. Finally, ghrelin‐treated C2C12 cells exposed to the specific CRF‐R2 ligand Ucn3 exhibited cAMP and pERK activation (Gershon & Vale, 2014). In this study, we explored the effects of des‐acyl ghrelin in C2C12 cells and demonstrate that it upregulates the signaling of CRF‐Rs. We further show that des‐acyl ghrelin affects the metabolism of C2C12 cells. Finally, we show that selective CRF‐R antagonists can reverse des‐acyl ghrelin's metabolic effects in C2C12 cells.

## 2 MATERIALS AND METHODS

### 2.1 Reagents

Des‐acyl ghrelin was purchased from Bachem. Ucn3, stressin, and antisauvagine‐30 were gifts from Dr. Jean Rivier (Salk Institute). Antalarmin was generously provided by Dr. G. Chrousos. The RNA extraction kit was purchased from QIAGEN. A High‐Capacity cDNA Synthesis Kit was purchased from Applied Biosystems. LightCycler 480 SYBR Green Master Mix for real‐time PCR was purchased from Roche. HotMaster Taq DNA polymerase was purchased from 5 PRIME. Acetyl‐CoA carboxylase (ACC) antibody and horseradish peroxidase‐linked secondary antibody were purchased from Cell Signaling. The antiactin antibody was purchased from AbCam. Oil red O, oleic acid, and palmitic acid were purchased from Sigma.

### 2.2 Cells

Cells of the mouse myoblast cell line C2C12 were obtained from the American Type Culture Collection. The cells were grown in Dulbecco's Modified Eagle Medium (DMEM; Invitrogen) with 10% fetal calf serum (FCS; Hyclone) at 37°C and 5% CO_2_. C2C12 cells were plated in a 12‐well Costar plate, allowed to recover for 24 hr, and then exposed to either des‐acyl ghrelin or vehicle in the medium for the indicated times. For treatment with CRF antagonists, the cells were plated and treated with des‐acyl ghrelin or vehicle as described above. Then the CRF‐R1‐ or CRF‐R2‐specific antagonists antalarmin and antisauvagine‐30, respectively, were added for 48 hr to the cell medium. The medium was refreshed every 24 hr. At the end of the incubation time, the cells were collected and examined as described in the following.

### 2.3 Glucose uptake by C2C12 cells

Glucose uptake by C2C12 cells was measured as described previously (Gershon & Vale, 2014). Briefly, after 2 hr incubation in low‐glucose medium and 2 hr incubation in Hank's balanced soil solution (HBSS) buffer, 10 nM insulin was added for 30 min incubation. Then, a mixture of [^3^H]‐deoxyglucose (0.2 mCi/ml) and nonradioactive 2‐deoxyglucose (0.1 mM) was added to the cells for an additional 5 min. At the end of the incubation, cells were washed with PBS and 1 M NaOH was added for 30 min. An aliquot of the sample was taken for protein determination and then the samples were neutralized using 1 M HCl. The extracts were counted for radioactivity in EcoLume scintillation fluid using a beta‐counter.

### 2.4 RNA extraction and RT‐PCR analysis

Total RNA was extracted from C2C12 cells using RNeasy mini columns (QIAGEN) according to the manufacturer's guidelines. RNA was converted into cDNA with the High‐Capacity cDNA Kit (Applied Biosystems) according to the manufacturer's guidelines using oligo (dT) and Moloney murine leukemia virus reverse transcriptase. The cDNA was used for quantitative PCR analysis, carried out on a StepOnePlus PCR system (Applied Biosystems) using Absolute Blue QPCR Master Mix (Thermo Scientific) with SYBR Green. The reaction protocol was as follows: 15 min at 95°C for enzyme activation, followed by 40 cycles of 15 s at 95°C, 30 s at 60°C, and 15 s at 72°C, at the end of which fluorescence was measured with the Rotor‐Gene PCR Cycler. SYBR Green I assays also included a melting curve at the end of the cycling protocol, with continuous fluorescence measurement from 65–99°C. All reactions contained the same amount of cDNA, 10 μl Absolute Blue QPCR Master Mix, primers for the indicated genes (Table 1) and UltraPure PCR‐grade water (Biological Industries) to a final volume of 20 µl. Each real‐time PCR included a no‐template control, in duplicate. Relative expression levels (ΔΔCt) were calculated by normalizing to hypoxanthine‐guanine phosphoribosyltransferase (*HPRT*). Primers were designed using the primer3 website (http://frodo.wi.mi‐ t.edu/primer3).

**TABLE 1:** List of primers used in this study

Gene	5′ Primer	3′ Primer	Accession number	Amplicon size (bp)
*GLUT4*	GATTCTGCTGCCCTTCTGTC	CAGCTCAGCTAGTGCGTCCAG	NM_009204	130
*RBP4*	GGAAACGATGACCACTGGAT	CATTGGGGTACGAGAAAAC	NM_001159487	130
*UCP2*	ACAGCCTTCTGCACTCCTG	GGCTGGGAGACGAAACACT	NM_011671	80
*UCP3*	TGCTGAGATGGTGACCTACG	CGGGTCTTTACCACATCCAC	NM_009464	149
*HPRT*	GCAGTACAGCCCCAAAATGG	GGTCCTTTTCACCAGCAAGCT	NM_13556	101

### 2.5 cAMP measurements

C2C12 cells were plated in a 24‐well Costar tissue dish with their growing medium and allowed to recover for 24 hr. Cells were treated with des‐acyl ghrelin or vehicle for 72 hr. At least 2 hr before treatment, the medium was changed to DMEM with 0.1% FBS. The cells were incubated with 0.1 mM 3‐isobutyl‐1‐methylxanthine for 30 min. The cells were then exposed to stressin or Ucn3 for an additional 30 min at 37°C. Intracellular cAMP concentrations were measured in triplicate using a cAMP radioimmunoassay kit (Biomedical Technologies).

### 2.6 Quantitative oil red O staining

C2C12 cells were incubated overnight in low‐glucose serum‐free medium. Cells were washed twice with HBSS buffer and incubated in high fatty acid media (200 µmol/L oleic acid, 100 µmol/L palmitic acid, and 0.5% bovine serum albumin) for an additional 18 hr, and then washed again with HBSS buffer and 3%oil red O solution was added to the cells. Following 1 hr incubation at room temperature, cells were washed twice with HBSS buffer and trypsin was added for 10 min at 37°C. Cells were harvested and DMEM with 10% FCS was added. After centrifugation at 3,000 rpmf or 5 min, the supernatant was removed and the pellet was rinsed with PBS. The pellet was resuspended in 70% ethanol and centrifuged for 1 min at 3,000 rpm. The supernatant was removed and its optical density was measured at 492 nm in triplicate. The pellet was then lysed and analyzed for total protein content using the Bradford method.

### 2.7 Western blot analysis

Total proteins were extracted from C2C12 cells, and Laemmli buffer (125 mM Tris, pH 6.8, 4% sodium dodecyl sulfate, 10% glycerol, 0.006% bromphenol blue, and 2% ß‐mercaptoethanol) was added. The samples were separated on a 12% acrylamide gel and transferred to a nitrocellulose membrane. Then, the membrane was incubated with a blocking solution (10% skimmed milk in Phosphate‐Buffered Saline, 0.1% Tween), followed by incubation with primary antibodies overnight at 4°C (dilution for ACC – 1:1,000, for β‐actin – 1:5,000 in 5% milk in TBS‐T). Then membranes were incubated with secondary antibodies (diluted 1:5,000 in 5% milk in TBS‐T) for 1 hr at room temperature. The immunoreactive bands were detected by enhanced chemiluminescence (Amersham, Buckinghamshire, England).

### 2.8 Immunofluorescence

Immunostaining was performed as described previously (Gershon & Vale, 2014). Briefly, C2C12 cells were plated in a 12‐well Costar plate. The cells were incubated in DMEM with 10% FBS for 24 hr after which ghrelin or vehicle was added for 72 hr. The cells were fixed with 4% paraformaldehyde for 20 min at room temperature, followed by the incubation in blocking solution (2% normal goat serum and 0.2% Triton X‐100 in PBS). Then the cells were incubated overnight at 4°C with anti‐GLUT4 primary antibody (dilution 1:100 in blocking solution). The next day, cells were washed three times with PBS and incubated at room temperature for 1 hr with Alexa 488‐conjugated anti‐rabbit secondary antibody (dilution 1:500 in blocking solution). After incubation, cells were mounted on slides with Vectastain mounting solution containing DAPI. Staining was visualized with a Zeiss LSM 710 laser scanning confocal microscope.

### 2.9 Statistical analysis

For statistical analysis, replicate experiments were averaged and analyzed by two‐way ANOVA. Differences were considered significant at *p* < .05. All numerical data shown in the figures are from representative experiments, expressed as means ± *SEM* of the replicates.

## 3 RESULTS

**FIGURE 1:**
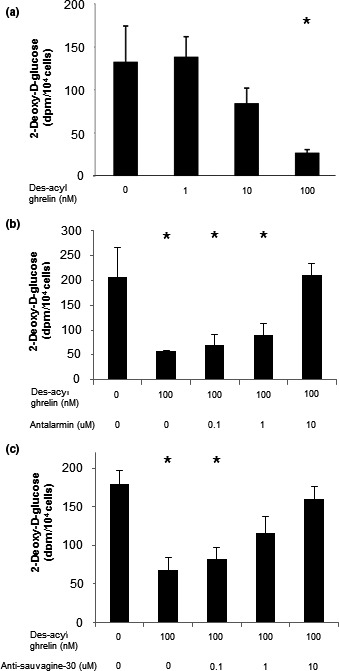
CRF‐R1 and CRF‐R2 inhibitors block des‐acyl ghrelin inhibition of glucose uptake. Des‐acyl ghrelin decreases H^3^‐deoxy‐glucose uptake into C2C12 cells (a). Antalarmin, a CRF‐R1‐specific antagonist (b), and antisauvagine‐30, a CRF‐R2‐specific antagonist (c), inhibit des‐acyl ghrelin‐induced glucose uptake. The examples shown are representative of at least three experiments. *p < .05 versus control

We have previously shown that ghrelin has an effect on muscle metabolism (Gershon & Vale, 2014). Des‐acyl ghrelin treatment dose‐dependently decreased glucose uptake in C2C12 cells (Figure 1a). Our previous study further demonstrated that both CRF‐R1 and CRF‐R2 expression levels are significantly increased upon exposure of C2C12 cells to des‐acyl ghrelin (Gershon & Vale, 2014). Furthermore, the addition of either antalarmin or antisauvagine‐30 (CRF‐R1‐ and CRF‐R2‐specific antagonists, respectively) to the medium of the cells during the last 24 hr of incubation reversed the des‐acyl ghrelin effect on glucose uptake into C2C12 cells (Figure 1b,c), suggesting that CRF‐R1 and CRF‐R2 mediate, directly or indirectly, glucose‐uptake induction by des‐acyl ghrelin in C2C12 cells.

**FIGURE 2:**
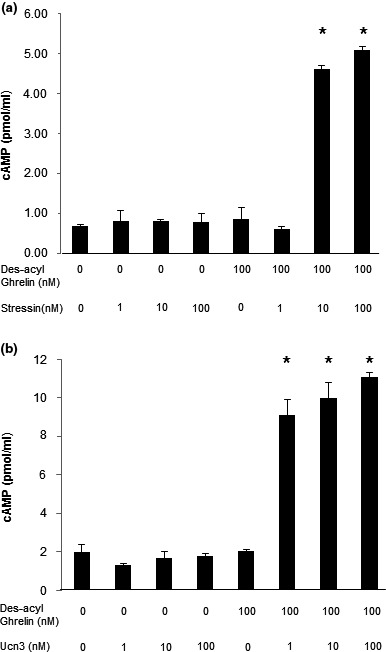
Des‐acyl ghrelin increases cAMP levels in C2C12 cells treated with CRF‐R1‐ or CRF‐R2‐selective ligands. (a) Stressin‐stimulated and (b) Ucn3‐stimulated cAMP accumulation in C2C12 cells pretreated with des‐acyl ghrelin. The examples shown are representative of at least three experiments. *p < .05 versus control

To determine whether the effect of des‐acyl ghrelin on the levels of CRF‐R1 and CRF‐R2 is modified by those receptors' own signaling, cAMP induction in response to either stressin or Ucn3, CRF‐R1‐ and CRF‐R2‐selective agonists, respectively, was assessed in des‐acyl ghrelin‐pretreated C2C12 cells. No significant difference was observed in cAMP levels between C2C12 cells treated with only des‐acyl ghrelin and untreated cells (Figure 2a,b). However, while stressin and Ucn3 did not modify cAMP levels in nontreated cells, accumulation of cAMP in C2C12 cells exposed to des‐acyl ghrelin was detected (Figure 2a,b). Furthermore, this stimulation was dependent on the doses of stressin and Ucn3 (Figure 2a,b). Based on this result, we concluded that des‐acyl ghrelin may have an impact on C2C12 cells' response to CRF‐R1 and CRF‐R2.

**FIGURE 3:**
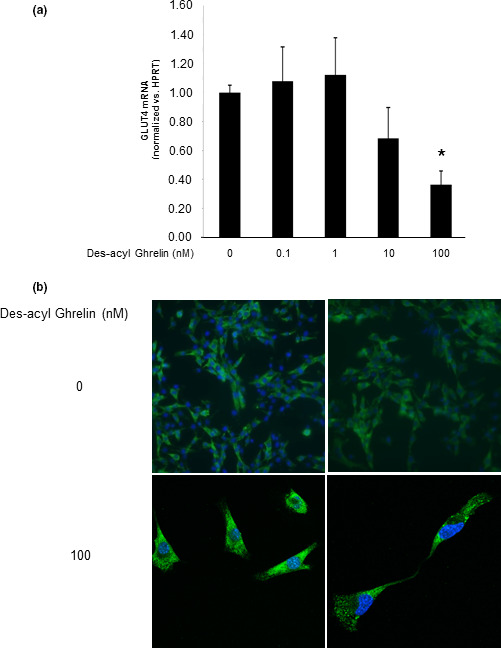
Des‐acyl ghrelin reduces GLUT4 levels in C2C12 cells. Des‐acyl ghrelin reduces GLUT4 mRNA (a) and protein (b) expression levels in C2C12 cells. The examples shown are representative of at least three experiments. *p < .05 versus control

**FIGURE 4:**
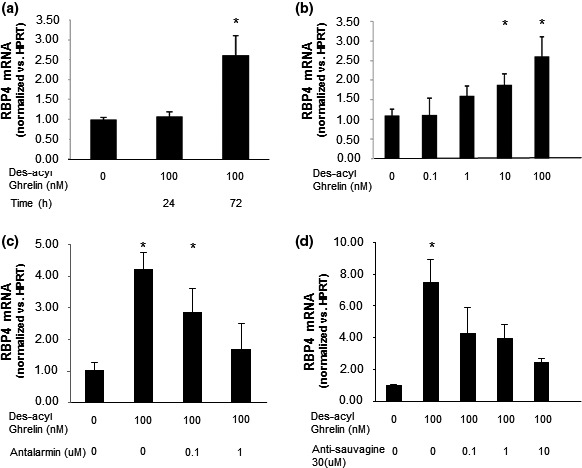
Des‐acyl ghrelin decreases *RBP4* levels in C2C12 cells. Prolonged exposure to des‐acyl ghrelin upregulates *RBP4* mRNA expression levels (a) in a dose‐dependent manner (b). Antalarmin, a CRF‐R1‐specific antagonist (c), and antisauvagine‐30, a CRF‐R2‐specific inhibitor (d), blocked this effect of ghrelin on *RBP4* mRNA levels. The examples shown are representative of at least three experiments. *p < .05 versus control

The most important protein regulating glucose uptake by muscle cells is GLUT4. A decrease in GLUT4 mRNA and protein levels was observed in response to des‐acyl ghrelin (Figure 3a,b, respectively). Exploring des‐acyl ghrelin's influence on the expression of RBP4, which has been shown to reduce insulin sensitivity, leading to diabetes, revealed that des‐acyl ghrelin treatment of C2C12 cells leads to a time‐ and dose‐dependent increase in the mRNA levels of *RBP4* (Figure 4a,b). As demonstrated for glucose uptake, the modification in *RBP4* levels induced by des‐acyl ghrelin could also be reversed by antalarmin or antisauvagine‐30, inhibitors of CRF‐R1 and CRF‐R2, respectively (Figure 4c,d). The expression levels of *RBP4* were not changed by treating C2C12 cells with either of these specific inhibitors alone (data not shown).

**FIGURE 5:**
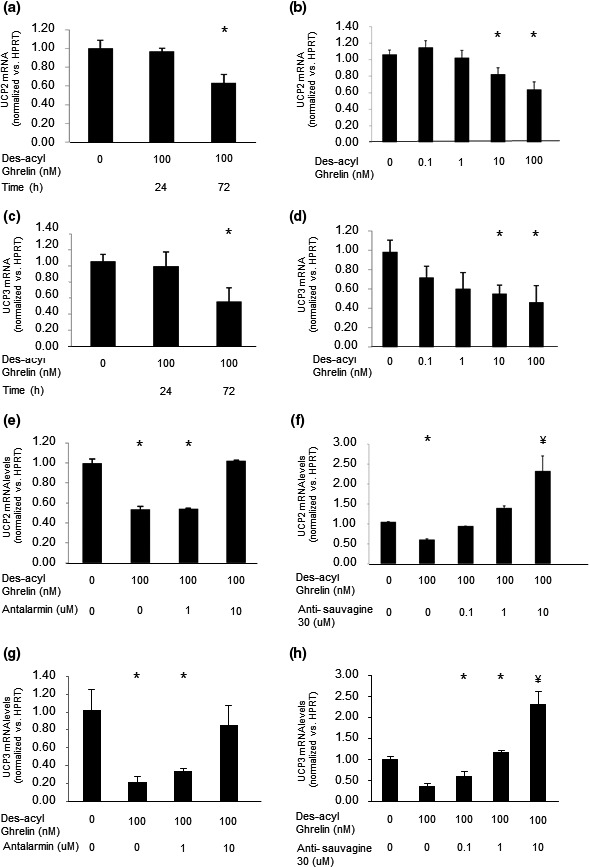
Ghrelin upregulates *UCP2* and *UCP3* mRNA expression in C2C12 cells. Prolonged exposure to ghrelin increases both *UCP2* and *UCP3* mRNA expression levels in a time‐dependent (a and c, respectively) and dose‐dependent (b and d, respectively) manner. The CRF‐R1‐specific antagonist antalarmin (e, g), and CRF‐R2‐specific inhibitor antisauvagine‐30 (f, h) blocked this effect of ghrelin on both *UCP2* and *UCP3* mRNA levels. The examples shown are representative of at least three experiments. *p < .05 versus control

We also found a time‐ and dose‐dependent decrease in *UCP2* (Figure 5a,b) and *UCP3* (Figure 5c,d) levels in response to des‐acyl ghrelin. This effect could also be reversed by antalarmin and antisauvagine‐30 (Figure 5e–h). The expression levels of *UCP2* and *UCP3* were not changed by treating C2C12 cells with either of these specific inhibitors alone (data not shown).

Although treating C2C12 cells with CRF‐R antagonists attenuated des‐acyl ghrelin's effects, it is important to remember that via its receptors, CRF has independent effects on those cells (Chen et al., 2006); therefore, treating C2C12 cells with the CRF‐R antagonists might have additional effects. These could explain the significantly higher mRNA levels of both *UCP2* and *UCP3* observed when treating the cells with a high concentration of antisauvagine‐30 (Figure 5f,h).

**FIGURE 6:**
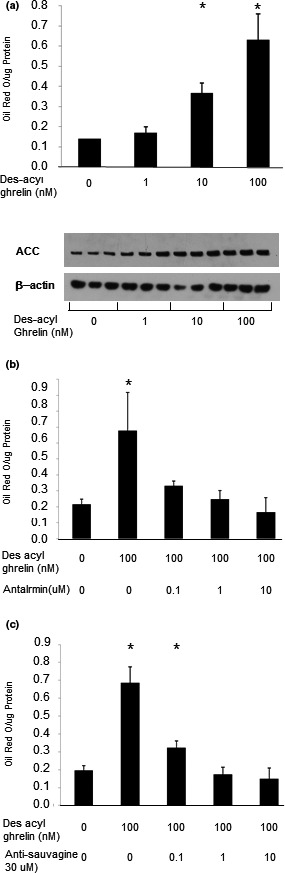
Des‐acyl ghrelin upregulates lipid content and fatty acid‐related protein expression. (a) Different concentrations of des‐acyl ghrelin were added to the cells for 72 hr, and lipid content assay was performed. (b) Cells were incubated with the indicated concentrations of des‐acyl ghrelin for 72 hr. Then proteins were extracted from the cells and ACC expression levels were examined. (c, d) C2C12 cells were incubated with des‐acyl ghrelin, and antalarmin (c) or antisauvagine‐30 (d) was added to the medium; lipid content was measured according to the protocol described in Materials and Methods. The examples shown are representative of at least three experiments. *p < .05 versus control

In another series of experiments, we investigated des‐acyl ghrelin's effects on fatty acid metabolism, another major energy source in muscle cells. Des‐acyl ghrelin elevated lipid content in the cells (Figure 6a). One of the enzymes that play a key role in fatty acid synthesis is ACC, and exposure of C2C12 cells to des‐acyl ghrelin increased its expression (Figure 6b). This increase in lipid content induced by des‐acyl ghrelin could be blocked by either antalarmin or antisauvagine‐30 (Figure 6c,d).

## 4 DISCUSSION

In this study, des‐acyl ghrelin decreased glucose uptake and increased lipid content in C2C12 cells. Furthermore, it significantly upregulated CRF‐R1 and CRF‐R2 expression levels. Our results with the selective antagonists of CRF‐R1 and CRF‐R2 suggest that the effects of des‐acyl ghrelin on glucose and lipid content are regulated by these two receptors. These results further raise the possibility that des‐acyl ghrelin plays a key role in regulating C2C12 functions. It is also suggested that excessive doses of des‐acyl ghrelin might hamper C2C12 cells' metabolic homeostasis. These results are in contrast to our previous observation that in C2C12 cells, ghrelin increases glucose uptake (Gershon & Vale, 2014). Taken together, these two studies offer a comparison of the effects of ghrelin versus des‐acyl ghrelin on C2C12 myotube cells, and support the notion of a possible relationship between the CRF family and ghrelin.

Our data suggest that high levels of des‐acyl ghrelin for an extended period of time inhibit the glucose metabolic pathway and increase fat utilization. Recent studies that support our findings have shown that in isolated muscles from rats, des‐acyl ghrelin increases fatty acid oxidation in vitro (Cervone et al., 2020; Kraft et al., 2019). Furthermore, under high fatty acid conditions, des‐acyl ghrelin protects isolated mature rat skeletal muscle from insulin resistance by activating the AMP‐activated protein kinase/ACC axis, resulting in increased oxidation of fatty acids (Cervone et al., 2020). It is important to note that while those studies found that des‐acyl ghrelin increases fatty acid oxidation (Cervone et al., 2020; Kraft et al., 2019), the present study found increased fat accumulation in C2C12 cells. Further studies are therefore needed to examine the effect of high levels of des‐acyl ghrelin for an extended period of time on fatty acid oxidation in C2C12 myoblasts.

In accordance with our results, ghrelin and des‐acyl ghrelin have opposing metabolic effects in cardiomyocytes (Lear et al., 2010). Nevertheless, the latter study showed that des‐acyl ghrelin, but not ghrelin, induces GLUT4 translocation to cell membranes and ghrelin inhibits, whereas des‐acyl ghrelin stimulates glucose uptake into the cells. The difference between our results and those studies may be due to the different types of muscle used and suggests that ghrelin isoforms have different effects on different muscle or cell types. In accordance with our data, Lear et al. (2010) found that des‐acyl ghrelin, but not ghrelin, increases fatty acid uptake to cardiomyocytes. This effect of ghrelin has also been demonstrated in adipocytes (Miegueu et al., 2011). Another paper showed that des‐acyl ghrelin increases adiposity both in vitro and in vivo (Heppner et al., 2014). Taken together, our data in this paper support previous studies showing that des‐acyl ghrelin is crucial for lipid metabolism in muscle cells.

Ghrelin has been shown to have effects on lipogenesis in human visceral adipocytes (Rodriguez et al., 2009). It has also been shown that des‐acyl ghrelin reduces lipolysis in both subcutaneous and visceral adipose tissue depots (Cervone et al., 2019). Both isoforms of ghrelin upregulated expression levels of transcription factors involved in promoting adipogenesis, such as PPARγ and SREBP1, as well as the expression of fat‐storage enzymes, including ACC, fatty acid synthase, lipoprotein lipase, and perilipin (Cervone & Dyck, 2017; Cervone et al., 2020; Theander‐Carrillo et al., 2006). Based on these results and our finding that des‐acyl ghrelin upregulates ACC expression and lipid content in the cells, we can conclude that des‐acyl ghrelin, in both adipocytes and C2C12 cells, causes an increase in intracytoplasmic lipid accumulation.

Previous reports have found that ghrelin affects glucose metabolism in the same manner in vivo. For example, mice lacking ghrelin expression showed higher glucose disposal (Tsubone et al., 2005). Furthermore, the sensitivity to glucose observed in this study might be a result of reduced expression of UCP2 (Tsubone et al., 2005). Ghrelin and leptin double‐knockout (KO) mice suffer from reduced glucose levels and increased insulin sensitivity (Miegueu et al., 2011). Other studies have shown that chronic infusion of ghrelin leads to an increase in the expression of UCP2 mRNA in white adipose tissue (Andrews et al., 2010), liver (Barazzoni et al., 2005), and pancreas (Tsubone et al., 2005). In addition, a significant elevation in both body fat and body weight was observed in UCP2‐KO mice after chronic i.p. ghrelin treatment (Yoshimoto et al., 2002). These results suggest a role for UCP2 in fat metabolism by restricting ghrelin‐induced lipogenesis. Although these results agree well with our data, Yoshimoto et al., (2002) could not determine whether ghrelin or des‐acyl ghrelin was responsible for the changes in glucose homeostasis, since mice lacking expression of the ghrelin gene lack both of its isoforms. Our in vitro studies with the C2C12 cell line suggested that des‐acyl ghrelin is the isoform that regulates glucose homeostasis in the periphery.

Our results further suggest that des‐acyl ghrelin enhanced fatty acid metabolism and reduced the level of UCP2 expression, leading to the induction of fat metabolism. Des‐acyl ghrelin also decreased glucose uptake by those cells. Thus, the overall action of des‐acyl ghrelin resulted in a shift of the cell's energy metabolism from glucose as the metabolic fuel to the fatty acid pathway. This suggested mechanism is supported by studies demonstrating that chronic ghrelin treatment induces body weight gain in wild‐type and *ucp2*‐KO mice; however, body weight gain is potentiated in *ucp2*‐KO mice (Andrews et al., 2010 #217). That study further demonstrated that the increase in body weight was solely a result of increased body fat due to decreased fat oxidation in *ucp2*‐KO mice, suggesting that UCP2 plays an important role in restricting ghrelin‐induced lipogenesis (Andrews et al., 2010). In addition, it has been shown that in rat myoblasts, ghrelin reduces the expression of UCP3 and palmitic acid‐induced triglyceride accumulation and prevents the palmitic acid‐induced decrease in glucose uptake in rat myoblasts (Han et al., 2015). On the other hand, in C2C12 cells, ghrelin downregulated fatty acid metabolism and favored glucose uptake (Gershon & Vale, 2014).

UCN administration to the rat hypothalamic paraventricular nucleus has been shown to result in a significant decrease in *UCP3* mRNA in the acromiotrapezius muscle (Kotz et al., 2002), suggesting that UCN has an effect on UCP expression in the muscle and on muscle metabolism. Our finding that administration of antisauvagine‐30 together with des‐acyl ghrelin led not only to block the effects of des‐acyl ghrelin on UCP2 and 3 expression, but it nearly doubled their expression, supports the notion that UCNs affect UCP expression in the muscle and muscle metabolism in a ghrelin‐dependent and ‐independent manner. Our results further suggest that those effects of UCNs in the muscle are mediated by CRF‐R2.

It has been shown that 90% of circulating ghrelin is in the nonacylated form (Kojima et al., 2004). Furthermore, des‐acyl ghrelin might act synergistically with, or antagonize ghrelin. In addition, des‐acyl ghrelin has ghrelin‐independent activity. However, it is still not clear whether des‐acyl ghrelin has its own receptor or whether it binds with a known ghrelin receptor (Delhanty et al., 2012; Zhang et al., 2017). Based on this knowledge, we can speculate that during hunger, when blood ghrelin levels increase, des‐acyl ghrelin is the dominant ghrelin isoform in the circulation. However, des‐acyl ghrelin has been shown to enhance insulin‐stimulated glucose uptake in rats (Gortan Cappellari et al., 2016). This result contradicts our data, a discrepancy that may be attributed to the use of myotubes, which are well‐differentiated muscle cells, in Gortan Cappellari et al.'s (2016) study, whereas we used C2C12 myoblasts. The fact that C2C12 myoblasts exclusively express CRF‐R1, whereas differentiated C2C12 myotubes primarily express CRF‐R2 (Kuperman et al., 2011), combined with our finding that des‐acyl ghrelin significantly upregulates CRF‐R1 levels (Gershon & Vale, 2014), support the suggested explanation and also raise the possibility that des‐acyl ghrelin upregulates the expression of CRF‐R1 in C2C12 cells.

A relationship between the CRF family and ghrelin isoforms has been previously demonstrated in the peripheral (Cervone et al., 2020; Chen et al., 2005) and central (Asakawa et al., 2001; Cabral et al., 2012; Kageyama et al., 2011; Kodomari et al., 2009; Tabarin et al., 2007) nervous systems. Here, we demonstrate that CRF‐R antagonists can block the effects of des‐acyl ghrelin on C2C12 cells, suggesting their similar role in muscle cell metabolism. The data suggest that the CRF family is regulated by the effect of des‐acyl ghrelin in the muscle, and not only in the central nervous system or on gut motility. Our results support the idea that the CRF family regulates or mediates the ghrelin isoforms' effects on muscle metabolism. A recent study supports our findings in showing that a decline in CRF‐R2 levels in soleus muscle isolated from high‐fat‐fed rats resulted in inhibition of the palmitate oxidation stimulated by ghrelin (Cervone et al., 2020).

Our published study (Gershon & Vale, 2014) and the data presented here support the notion that CRF‐R2 is a downstream effector of ghrelin and des‐acyl ghrelin which might be involved in mediating both ghrelin isoforms' effects on muscle metabolism. The ability of CRF‐R antagonists to block the effects of des‐acyl ghrelin on RBP4 expression in C2C12 cells provides additional support for02 the relationship between CRF‐Rs and ghrelin, as does a study showing that skeletal muscles isolated from CRF‐R2‐null mice express significantly less RBP4 compared to the wild type (Kuperman et al., 2011).

Elevated levels of ghrelin isoforms are detected in many diseases, including eating disorders (Krsek et al., 2003), the development of type 2 diabetes and anorexia nervosa (Karczewska‐Kupczewska et al., 2010; Kojima et al., 2004). Understanding the actions and relationships of ghrelin and des‐acyl ghrelin with muscle cell development and function will help elucidate their mechanisms in muscle metabolism, under normal and pathological conditions. Our data further suggest that in high‐ghrelin states, the CRF‐Rs' suppressed activity might be valuable.

## CONFLICT OF INTEREST

None of the authors have any potential conflicts of interest associated with this research.

## AUTHORS' CONTRIBUTIONS

ME and EG conceived the study, designed the experiments, performed the experiments, and analyzed data. ME drafted the manuscript. EG revised the manuscript. All authors approved the final manuscript.
